# Supplementary material for: Vitamin D Modified DSS-Induced Colitis in Mice via STING Signaling Pathway
Source: Biology (Basel). 2025 Jun 18;14(6):715. doi: 10.3390/biology14060715 (PMC12190092; doi:10.3390/biology14060715)
Supplement: Supplementary file 1 [file biology-14-00715-s001.zip › Supplementary Materials S1: 16s-report of gut microbiota/Groups/heatmap/Group/norm_heatmap_log10.pdf]

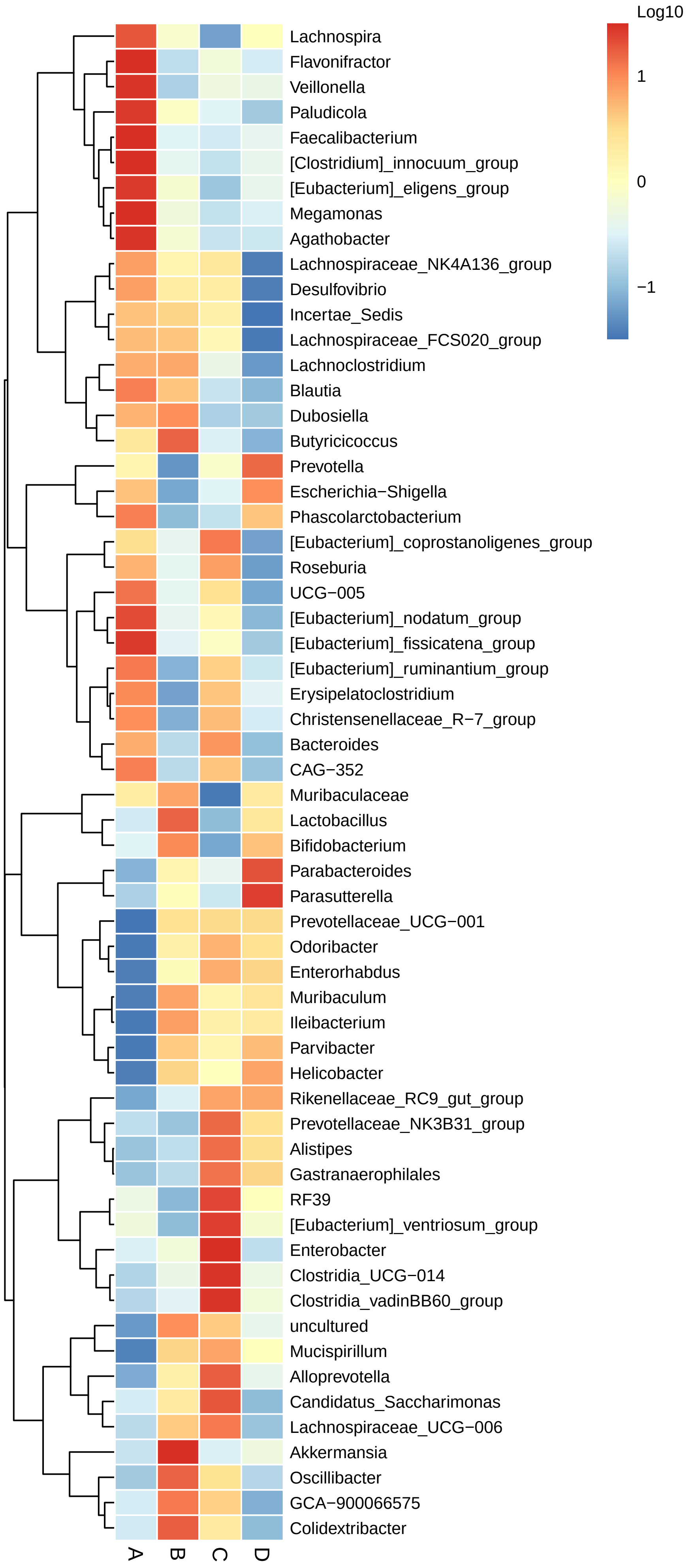

Log10
1
0
−1
Lachnospira
Flavonifractor
Veillonella
Paludicola
Faecalibacterium
[Clostridium]_innocuum_group
[Eubacterium]_eligens_group
Megamonas
Agathobacter
Lachnospiraceae_NK4A136_group
Desulfovibrio
Incertae_Sedis
Lachnospiraceae_FCS020_group
Lachnoclostridium
Blautia
Dubosiella
Butyricicoccus
Prevotella
Escherichia−Shigella
Phascolarctobacterium
[Eubacterium]_coprostanoligenes_group
Roseburia
UCG−005
[Eubacterium]_nodatum_group
[Eubacterium]_fissicatena_group
[Eubacterium]_ruminantium_group
Erysipelatoclostridium
Christensenellaceae_R−7_group
Bacteroides
CAG−352
Muribaculaceae
Lactobacillus
Bifidobacterium
Parabacteroides
Parasutterella
Prevotellaceae_UCG−001
Odoribacter
Enterorhabdus
Muribaculum
Ileibacterium
Parvibacter
Helicobacter
Rikenellaceae_RC9_gut_group
Prevotellaceae_NK3B31_group
Alistipes
Gastranaerophilales
RF39
[Eubacterium]_ventriosum_group
Enterobacter
Clostridia_UCG−014
Clostridia_vadinBB60_group
uncultured
Mucispirillum
Alloprevotella
Candidatus_Saccharimonas
Lachnospiraceae_UCG−006
Akkermansia
Oscillibacter
GCA−900066575
Colidextribacter
A
B
C
D
